# Plant-Specific AtS40.4 Acts as a Negative Regulator in Abscisic Acid Signaling During Seed Germination and Seedling Growth in *Arabidopsis*

**DOI:** 10.3389/fpls.2021.622201

**Published:** 2021-02-04

**Authors:** Xiao-Pu Shi, Jing-Jing Ren, Hao-Dong Qi, Yi Lin, Yu-Yi Wang, De-Feng Li, Lan-Jing Kong, Xiu-Ling Wang

**Affiliations:** ^1^National Key Laboratory of Crop Biology, College of Life Sciences, Shandong Agricultural University, Tai’an, China; ^2^Biology and Food Engineering School, Fuyang Normal University, Fuyang, China; ^3^Shandong Lufeng Group Co., Ltd., Anqiu, China

**Keywords:** *Arabidopsis thaliana*, *AtS40.4*, DUF584, abscisic acid signaling, *ABI4*, seed germination, early seedling growth

## Abstract

Abscisic acid (ABA) is an important phytohormone regulating plant growth, development and stress responses. A multitude of key factors implicated in ABA signaling have been identified; however, the regulation network of these factors needs for further information. AtS40.4, a plant-specific DUF584 domain-containing protein, was identified previously as a senescence regulator in *Arabidopsis*. In this study, our finding showed that AtS40.4 was negatively involved in ABA signaling during seed germination and early seedling growth. *AtS40.4* was highly expressed in seeds and seedlings, and the expression level was promoted by ABA. AtS40.4 was localized both in the nucleus and the cytoplasm. Moreover, the subcellular localization pattern of AtS40.4 was affected by ABA. The knockdown mutants of *AtS40.4* exhibited an increased sensitivity to ABA, whereas the overexpression of *AtS40.4* decreased the ABA response during seed germination and seedling growth of *Arabidopsis*. Furthermore, AtS40.4 was involved in ABRE-dependent ABA signaling and influenced the expression levels of *ABA INSENTIVE* (*ABI*)*1-5* and *SnRK2.6*. Further genetic evidence demonstrated that *AtS40.4* functioned upstream of *ABI4.* These findings support the notion that AtS40.4 is a novel negative regulator of the ABA response network during seed germination and early seedling growth.

## Introduction

Abscisic acid (ABA) is a key phytohormone, which plays versatile functions in developmental regulation and various environmental stress response. The ABA plays roles in spatiotemporal and dose-dependent ways, which is connected with its diverse functions (Liu et al., 2019; Lu et al., 2019; Chandrasekaran et al., 2020; Xie et al., 2020). ABA is well-known for its stress-related properties, but it also has been proven to regulate plant growth and development such as seed germination, root growth and flowering transition (Huang et al., 2017; Belda-Palazon et al., 2018; Xiong et al., 2019). ABA content in seeds rapidly decreases after imbibition, which facilitates seed germination; whereas the exposure of seeds to ABA during germination rapidly arrests seed growth (Zhu et al., 2011; Ye et al., 2012). ABA maintains or represses the primary root elongation and the lateral root formation during plants response to different environmental stimuli such as water deficit, osmotic and salt stress (Spollen et al., 2000; Zhang et al., 2010; Duan et al., 2013; Jiang et al., 2013). Recent researches reveal that ABA also regulate plant flowering time. Several core components of ABA signaling modulate expression levels of key genes in flowering transition (Shu et al., 2018; Conti, 2019; Xiong et al., 2019).

Many signaling details of ABA have been well-elucidated. ABA signaling network is tightly regulated and modulated by various factors at transcription and post-transcription levels in response to endogenous and exogenous cues (Yu et al., 2019; Ali et al., 2020; Gong et al., 2020). A subset of key genes involved in ABA signaling has been identified. Among these genes, *ABA INSENTIVE* (*ABI*) *1*, *ABI2*, *ABI3*, *ABI4*, and *ABI5*, encode five key negative or active regulators of ABA response (Leung et al., 1997; Skubacz et al., 2016; Chandrasekaran et al., 2020). *ABI1* and *ABI2* encode type 2C protein phosphatases (PP2Cs) that play roles as negative regulators of ABA signaling. PP2Cs interact with and inactivate ABA-activated sucrose non-fermenting 1-related protein kinase 2 (SnRK2) members, a core kinase in ABA signaling (Leung et al., 1997). ABI3, ABI4, and ABI5 are transcription factors (TFs) of the B3, APETALA2, and basic leucine zipper families, respectively, and share overlapping functions in ABA signaling as positive regulators (Finkelstein et al., 2002; Cheng et al., 2014; Liu et al., 2017; Chandrasekaran et al., 2020).

In addition to these TFs as trans-acting factors, there are several important cis-acting elements have been identified and well-characterized in ABA signaling such as ABA-responsive element (ABRE), coupling element and dehydration-responsive element (DRE) (Narusaka et al., 2003; Min et al., 2020). ABREs are major cis-acting regulators in ABA-dependent gene expression (Kim et al., 2004; Wang et al., 2019; Chen et al., 2020). ABI3, ABI4, and ABI5 bind directly to the ABREs of the promoter regions of a subset of genes, thereby controlling their transcription levels (Carles et al., 2002; Ezcurra et al., 2000; Mizoi et al., 2012; Shu et al., 2013; Skubacz et al., 2016; Wang et al., 2019; Chandrasekaran et al., 2020; Collin et al., 2020). Notably, ABI3, ABI4, and ABI5 either activate or repress gene expression in diverse processes depending on DNA binding. For instance, ABI4 directly or indirectly induces expression of genes involved in seed maturation; whereas represses transcription of genes functioning in photosynthesis and pigment metabolism, or ABA catabolic pathways (Wind et al., 2013; Baek et al., 2020; Khan et al., 2020). Considering that the core position of these ABIs in ABA signaling, it is very important to understand the expression regulation of *ABIs* genes.

The plant-specific senescence 40 (S40) family proteins that have a molecular weight of 12–25 kD and contain a plant-specific domain of unknown function 548 (DUF548) are widely present in plants (Fischer-Kilbienski et al., 2010; Jehanzeb et al., 2017; Zheng et al., 2019). DUF548 is an intriguing C-terminal domain sharing the sequence GRXLKGR(D/E) (L/M)XXXR(D/N/T)X(I/V)XXXXG(F/I) which is highly conserved in plant species (Jehanzeb et al., 2017). A subset of genes encoding DUF548 proteins have been identified in barley, *Arabidopsis* and rice (Krupinska et al., 2002; Fischer-Kilbienski et al., 2010; Zheng et al., 2019). Notably, multiple members of *S40* genes exist in one plant species. For example, there are 15 *S40s* (*AtS40s*) in *Arabidopsis* and 16 *S40s* (*OsS40s*) in rice (Fischer-Kilbienski et al., 2010; Jehanzeb et al., 2017). *AtS40s*, *OsS40s*, and barley *S40* (*HvS40*) participate in regulation of natural and stress-reduced leaf senescence (Krupinska et al., 2002; Fischer-Kilbienski et al., 2010; Janack et al., 2016; Jehanzeb et al., 2017; Zheng et al., 2019). AtS40.3, HvS40, OsS40-1, OsS40-13, and OsS40-14 are partly or completely localized in the nucleus. Moreover, AtS40.3 and HvS40 are assigned as DNA binding proteins (Krupinska et al., 2002; Fischer-Kilbienski et al., 2010). Interestingly, *AtS40.3* also affects the sex ratio of cyst nematodes in *Arabidopsis* (Anwer et al., 2018). Until now, the regulation of expression of these *S40* genes, the subcellular localization of these DUF548 proteins and their molecular mechanisms remain largely uncovered.

*AtS40.4* (At2G28400) encodes a cytoplasmic S40 protein and is associated with natural and darkness-induced senescence (Fischer-Kilbienski et al., 2010). *AtS40.4* expression is regulated by dark-induced senescence, salicylic acid and ABA treatments, and pathogen infections in rosette leaves of 4 weeks old plants. In this study, we showed that *AtS40.4* functions in seed germination and early seedling growth as a negative regulator in the ABA signaling pathway. AtS40.4 was localized both in the nucleus and the cytoplasm, and the localization pattern of AtS40.4 was affected by ABA. Moreover, AtS40.4 affects the expression of *ABIs* and ABRE-containing genes. These findings provide a new insight to understand the molecular features of these plant-specific DUF584 proteins in modulating ABA signaling.

## Materials and Methods

### Plant Materials and Growth Conditions

The material used in the experiment was *Arabidopsis thaliana* (Columbia, Col-0). Seeds of T-DNA insertion lines, *ats40.4-1* (GABI_561G08) and *ats40.4-2* (GABI_863C05), were obtained from the *Arabidopsis* Biological Resources Center (ABRC). Seeds were surface-sterilized, and placed on 0.5 × MS solid medium with or without ABA (Shi et al., 2018). After stratification for 3 days at 4°C in the dark to break dormancy, the plates were incubated at 20–22°C with a 16/8 h light/dark cycle. The genotyping was identified by using PCR with primers listed in Supplementary Table 1.

### Generation of Transgenic Plants and Double Mutant

To generate *35S:AtS40.4* construct, the full-length coding sequence (CDS) of *AtS40.4* was amplified using the specific primers (Supplementary Table 2) and was subcloned into the *pMD18-T* vector (Takara). Subsequently, the fragment was ligated into the binary vector *pBI121* by restriction enzymes *Xba*I and *Kpn*I, resulting in the *35S:AtS40.4* construct. For *proAtS40.4:GUS* construct, a 1,403 bp genomic fragment upstream of the ATG start codon of *AtS40.4* was cloned into the Gateway Entry vector (pCR8/GW/TOPO vector, Invitrogen) via gateway technology, and subsequently was introduced into the pMDC163 vector using the LR Clonase II Plus enzyme (Invitrogen) as described by Xiong et al. (2019). A promoter region (1,403 bp upstream of the start codon) with gene region of *AtS40.4* (489 bp with mutation of termination codon TAA to TAT) was subcloned into the pMDC107 vector via gateway technology (Invitrogen) to generate the *proAtS40.4:AtS40.4-GFP* construct. These constructs were introduced into *Agrobacterium tumefaciens* strain GV3101 and transformed into wild-type (*proAtS40.4:GUS* and *35S:AtS40.4*) or *ats40.4-1* mutant plants (*proAtS40.4:AtS40.4-GFP*) using the floral dip method (Clough and Bent, 1998). The transgenic seedlings were selected on 0.5 × MS medium containing 25 mg/L hygromycin or kanamycin and then identified by RCR using the primers (Supplementary Table 2). We used T3 seeds for further study.

The *abi4 ats40.4* double mutant was created by genetic crossing. The *abi4* (SALK_080095) mutant was described previously (Shu et al., 2013). We created the *6xABRE_A:erGFP ats40.4-1* plants by crossing *6xABRE_A:erGFP* into *ats40.4-1* background to mark the expression of *ABRE*-containing genes (Wu et al., 2018).

### Phenotypic Analysis

Phenotypic analysis was performed as described previously (Li Z. et al., 2016; Shi et al., 2018). Sterilized seeds of wild type, *ats40.4* mutants and *AtS40.4*-overexpressing lines were plated on ABA (Sigma-Aldrich) medium. For root elongation assays, the seedlings grown on 0.5 × MS medium for 3 days were transferred to new 0.5 × MS medium or medium containing ABA for further growth on vertical plates. All the results were calculated based on at least three replicate experiments, and more than 60 seeds or seedlings for each line were used for the quantification.

### Gene Expression Analysis

Gene expression analysis was performed by the reverse transcription- polymerase chain reaction (RT-PCR) and real-time quantitative RT-PCR (qRT-PCR) with the specific primers (Supplementary Tables 3, 4). Total RNA was isolated from samples with RNAprep pure Plant Kit (TIANGEN, DP441) and was used for reverse transcription using PrimeScript^TM^ RT reagent Kit (TAKARA). *TUBULIN2* and *GAPDH* were used as the control genes for RT-PCR and qRT-PCR, respectively. All experiments were performed from three biological replicates.

For analysis of expression levels of genes after ABA treatment, 5-day-old seedlings grown on 0.5 × MS liquid medium were used to exogenous ABA treatment. The seedlings were placed into 0.5 × MS liquid medium with ABA (10 μM, in ethanol), and the same amount of ethanol as a negative control. Only the roots of seedlings were immersed in ABA medium and whole seedlings were used for extraction of total RNA (Xiong et al., 2019). The expression levels of genes selected in ABA signaling were detected as descripted by Mei et al. (2014).

### GUS Histochemical Staining

Six independent lines expressing the *proAtS40.4:GUS* construct in Col-0 background were analyzed for GUS histochemical staining. GUS staining was performed as described by Xiong et al. (2019). Samples were fixed using 90% acetone for 20 min and then were immersed in the staining solution were incubated in staining solution (50 mM NaPO_4_, pH 7.2, 2 mM X-gluc, 0.5 mM K_3_Fe (CN)_6_, and 0.5 mM K_4_Fe (CN)_6_, 0.1% Triton X-100) at 37°C overnight after vacuum infiltration for 10 min on ice. Then stained materials were washed with 30, 50, 75, 85, 95, 100% ethanol for 1 h each to remove the chlorophyll.

To analyze the effect of ABA on *AtS40.4* expression, 3-day-old seedings transformed *proAtS40.4:GUS* were transferred into 0.5 × MS liquid medium with or without 10 μM ABA for 6 h before GUS staining.

### Fluorescence Imaging and Quantification

To explore the subcellular localization of AtS40.4, six independent lines transformed *proAtS40.4:AtS40.4-GFP* construct in *ats40.4-1* plants were selected. The green fluorescence in root tip cells of 7-day-old seedlings were detected. To test the effects of ABA on the subcellular localization of AtS40.4, the roots of 7-day-old seedlings were treated with 0.5 × MS liquid medium containing 100 μM ABA for 2 or 4 h (Xu et al., 2019). PEG-induced moderate severity (-0.75 MPa) was used to simulate drought stress to roots (Verslues et al., 2006; Chong et al., 2019).

The fluorescence signals of green fluorescent protein (GFP) and 4′,6-diamidino-2-phenylindole (DAPI) were captured using an LSM 880 laser scanning confocal microscope (Carl Zeiss) with the 488/505–550 and 358/460 nm excitation/emission wavelengths, respectively. Quantification of fluorescence intensity was performed using more than 30 plants in each sample. ImageJ was used to quantify the GFP fluorescence intensity.

## Results

### *Ats40.4* Mutations Increased Sensitivity to ABA During Seed Germination and Early Seedling Growth

**FIGURE 1:**
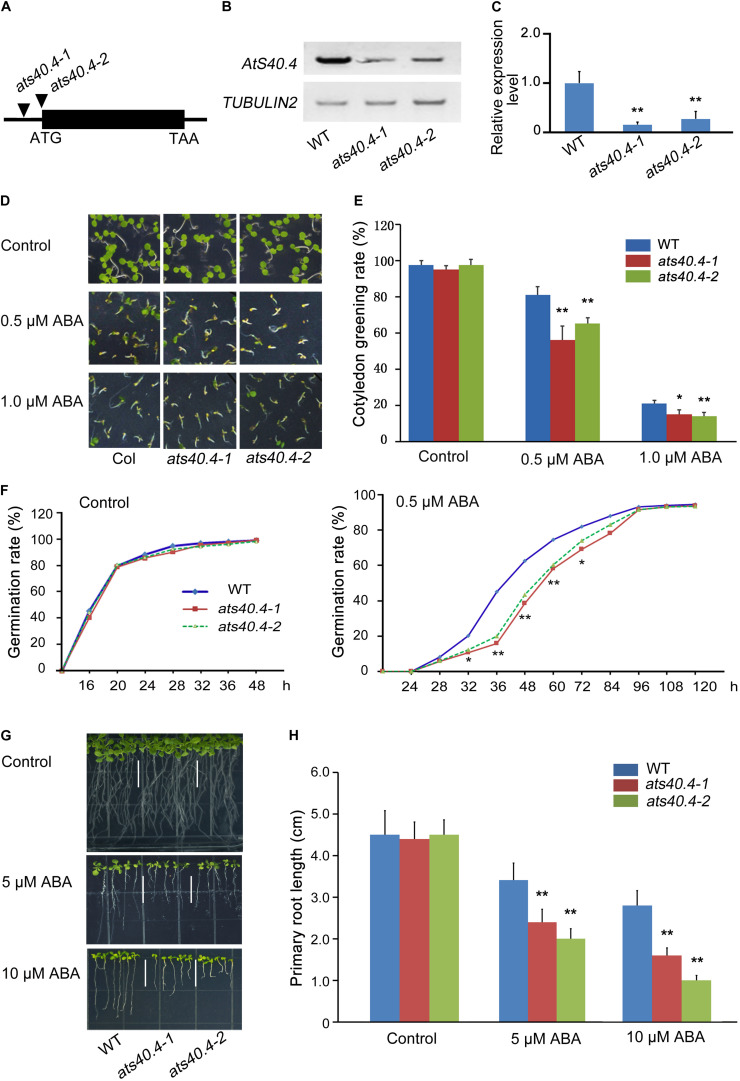
*AtS40.4* negatively regulates ABA signaling during seed germination and early seedling growth. **(A)** Genomic structure of *AtS40.4* (At2g28400) and the positions of T-DNA insertions (triangles) of *ats40.4-1* (GABI_561G08) and *ats40.4-2* (GABI_863C05). **(B,C)** RT-PCR and qRT-PCR analysis of *AtS40.4* expression in the seedlings of Col-0 wild-type (WT) and *ats40.4* mutants. *TUBULIN2* and *GAPDH* were used as internal controls for RT-PCR **(B)** and qRT-PCR **(C)**, respectively. Significant differences are denoted with double asterisks **(C)** (Student’s *t*-test, **P < 0.01). **(D)** Seed germination and seedling growth of the WT and *ats40.4* mutants grown on 0.5 × MS medium containing 0, 0.5, and 1.0 μM ABA. **(E)** Cotyledon greening rates of seedlings grown on 0.5 × MS medium containing 0, 0.5, and 1.0 μM ABA. Error bars indicate the SEs of three replicated experiments. Single and double asterisks indicate a significant difference (Student’s *t*-test; *P < 0.05; **P < 0.01). **(F)** Seed germination rates of WT and *ats40.4* mutants. Seeds were grown on 0.5 × MS medium containing 0 (left) and 0.5 μM ABA (right). Asterisks indicate a significant difference from the control value (*P < 0.05; **P < 0.01). **(G,H)** Comparison of root elongation of WT with *ats40.4* mutants. The 3-day-old seedlings grown on 0.5 × MS medium were transferred onto the medium containing 0, 5 and 10 μM ABA for further growth 14 days **(G)**. Data **(H)** are means ± SEs based on three biological replicates. Double asterisks indicate statistically significant differences between the mutants and WT calculated by Student’s *t*-test (**P < 0.01).

To understand the roles of these plant-specific genes which encode DUF-containing proteins in ABA signaling, we screened the *Arabidopsis* T-DNA insertional population with different responses to exogenous ABA from the wild type. Among these mutants, GABI_561G08 and GABI_863C05 (named as *ats40.4-1* and *ats40.4-2*), two mutants of *AtS40.4* (At2G28400), showed increased sensitivity to ABA during seed germination and seedling growth. The T-DNA insertion in the *AtS40.4* 5’ UTR in *ats40.4-1* and *ats40.4-2* mutants led to a significantly reduced expression of *AtS40.4* (Figures 1A–C).

*AtS40.4* encodes a DUF584 protein involved in the leaf senescence (Fischer-Kilbienski et al., 2010). To investigate the effect of *AtS40.4* on the ABA responses during seed germination and seedling growth, we analyzed the changes of seed germination and seedling growth of *ats40.4* mutants. In the absence of exogenous ABA, *ats40.4-1* and *ats40.4-2* mutants did not exhibit apparent difference during seed germination and seedling growth (Figure 1D). However, these *ats40.4* mutants displayed a significantly increased sensitivity of seed germination and cotyledon greening after ABA application (Figures 1E,F). Moreover, the root growth of the *ats40.4-1* and *ats40.4-2* mutants was more inhibited than that of wild-type seedlings by ABA (Figures 1G,H). The ABA hypersensitivity of *ats40.4-1* mutant was significantly decreased by expressing the *AtS40.4* CDS under the control of its native promoter (*proAtS40.4*:*AtS40.4-GFP*) (Supplementary Figures 1A–D), indicating that the disruption of the *AtS40.4* causes the ABA-hypersensitive phenotype.

To further confirm that *AtS40.4* functions in the ABA response during seed germination and seedling growth, we then overexpressed *AtS40.4* CDS in the wild-type *Arabidopsis* under the control of CaMV 35S promoter (*35S:AtS40.4*). The transcription level of *AtS40.4* in the transgenic lines (referred to as OE-1, OE-2, and OE-3) was 3–7-fold higher than that in the wild-type seedlings (Supplementary Figure 2A). The overexpression of *AtS40.4* decreased the sensitivity of seed germination and cotyledon greening to exogenous ABA (Supplementary Figures 2B–D). The primary roots of the *AtS40.4* overexpressing lines were significantly longer than those of the wild type (Supplementary Figures 2E,F).

Abiotic stresses stimulate the accumulation of endogenous ABA and multiple proteins are involved in the response of plants to abiotic stress through ABA-dependent way (Quesada et al., 2000; González-Guzmán et al., 2002; Lu et al., 2019). To investigate whether or not *AtS40.4* plays a role in abiotic stress-induced ABA signaling, we tested the seed germination of *ats40.4* mutants and *AtS40.4-*overexpressing lines on 0.5 × MS medium containing 175 mM NaCl. The germination rate was decreased in the *ats40.4* mutants, while increased in the *AtS40.4-*overexpressing lines under NaCl stress (Supplementary Figures 3A,B), indicating that *AtS40.4* might function in slat-induced ABA effects on seed germination. Altogether, these results indicate that *AtS40.4* participates in the ABA signaling pathway during seed germination and early seedling growth as a negative regulator.

**FIGURE 2:**
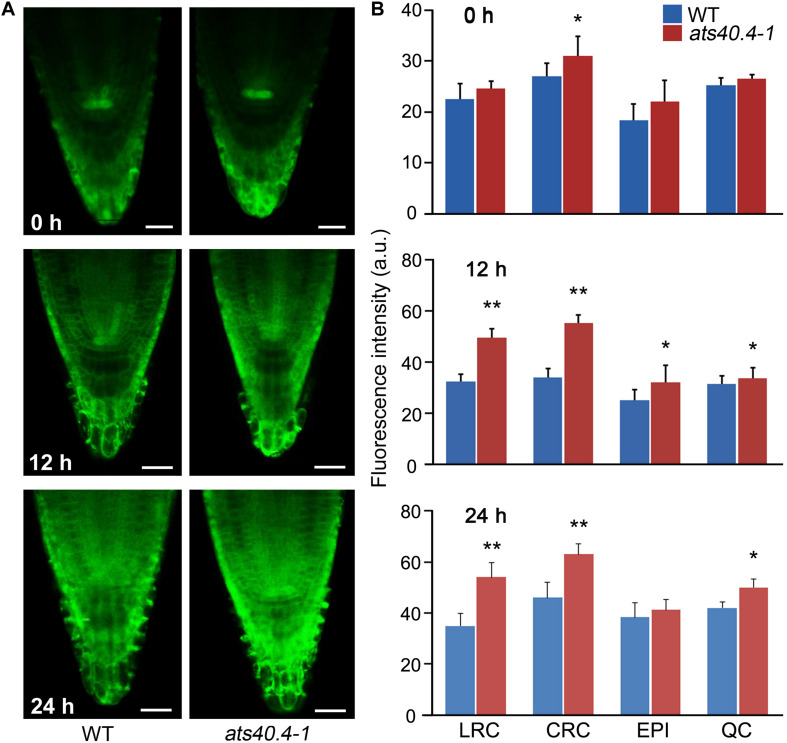
*AtS40.4* is involved in the ABRE-dependent ABA signaling. **(A)** Expression pattern of 6xABRE_A:erGFP in root tips of wild type and *ats40.4* background plants under 10 μM ABA applications for 0, 12 and 24 hours. **(B)** Quantified fluorescence of 6xABRE_A:erGFP in lateral root cap (LRC), columella root cap (CRC), epidermis (EPI) and quiescent center (QC) cells of root tip. Scale bars, 20 μm.

The ABRE is a major cis-element for ABA-responsive gene expression (Nakashima and Yamaguchi-Shinozaki, 2013; Gong et al., 2020). The 6 × ABRE_A:erGFP is an ABA responsive reporter that can mark the transcriptional response of tissues to ABA (Wu et al., 2018). To further investigate the *AtS40.4* functions in the ABA-mediated transcriptional regulation and the sites of action, we introduced *6* × *ABRE_A:erGFP* into the *ats40.4-1* plants and investigated the GFP fluorescence intensity and patterns. ABA application significantly enhanced the GFP-fluorescence intensity in the lateral root cap, columella root cap, epidermis and quiescent center cells in root tips of the seedlings (Figure 2), consisting with the previous report by Wu et al. (2018). Notably, the fluorescence intensity of GFP was significantly increased in the root cap cells of *ats40.4-1* background seedlings compared to those in the wild-type *Arabidopsis* after 10 μM ABA treatment for 12, or 24 h, indicating that AtS40.4 might function in the root cap to sense and response the soil conditions in ABA signaling during seedling growth. This supports that AtS40.4 is involved in ABRE-dependent ABA signaling.

### *Ats40.4* Is a Ubiquitously Expressed Gene and Highly Expressed in the Seeds and Seedlings

**FIGURE 3:**
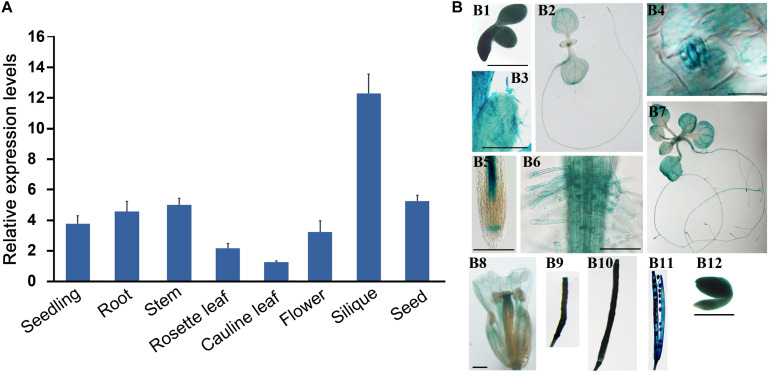
The expression patterns of *AtS40.4* in *Arabidopsis.* **(A)** Expression level of *AtS40.4* in *Arabidopsis* including seedlings, roots, stems, rosette leaves, cauline leaves, flowers, siliques, and developing seeds detected by qRT-PCR using the specific primers for *AtS40.4*. *GAPDH* was used as an internal control. Error bars indicate the SEs of three experimental replicates. **(B)** GUS histochemical staining shows the expression patterns of *AtS40.4* in *Arabidopsis* expressing the *proAtS40.4:GUS* construct. The strong GUS signal was observed in the germinating seed (B1), 2-day-old seedling (B2), guard cell (B3) and trichomes (B4) in leaf, root tip (B5), and root hairs (B6) of 2-week old seedling (B7), flower (B8), young (1 and 2 weeks after pollination, B9 and B10) and old-yellowing siliques (B11), and cotyledons of mature seeds (B12). The experiment was repeated twice with a similar result. Scale bars, 100 μm.

The participation of *AtS40.4* in ABA response during seed germination and seedling growth suggested that this gene may play roles at the early stages of *Arabidopsis* development besides the senescence leaves as described previously (Fischer-Kilbienski et al., 2010). We prepared the total RNA from the seedlings and roots, stems, rosette and cauline leaves, flowers, old-yellowing siliques, and mature seeds of *Arabidopsis* to examine the expression levels of *AtS40.4*. The qRT-PCR result showed that *AtS40.4* was expressed highly in the seedlings, stems, roots, siliques, and seeds (Figure 3A).

To further investigate the temporal and spatial expression patterns of *AtS40.4* in *Arabidopsis*, we generated a *proAtS40.4:GUS* construct, and transformed this construct into the Col-0 *Arabidopsis*. Four independent T3 transgenic lines were used to investigate the *AtS40.4* expression patterns during *Arabidopsis* development. The high GUS activity was detected in the cotyledons of 24 h imbibed seeds, 7-day- and 2-week-old seedlings; rosette and cauline leaves, stem, inflorescences, young and old-yellowing siliques, and cotyledons of mature seeds (Figure 3B). The trichomes and guard cells of the leaves, root tips and root hairs, and old-yellowing siliques exhibited strong GUS staining signals. These results demonstrated that *AtS40.4* is globally expressed in *Arabidopsis* at the vegetative growth and reproductive stages.

### The Gene Expression and Subcellular Localization of Ats40.4 Are Regulated by ABA

**FIGURE 4:**
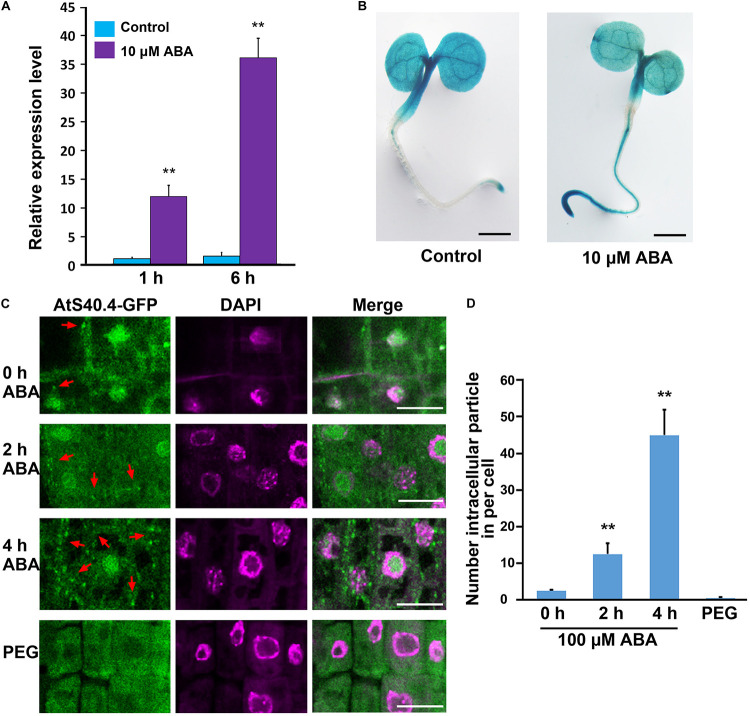
ABA affects *AtS40.4* expression and protein localization. **(A)** qRT-PCR analysis of *AtS40.4* mRNA level in seedlings. The 5-day-old seedlings grown on 0.5 × MS medium were cultured with free and 10 μM ABA treatment for 1 and 6 h. *GAPDH* was used as an internal control. Data are means ± SEs of three biological replicates. Double asterisks indicate significant differences (Student’s *t*-test; **P < 0.01). **(B)** GUS activity of transgenic *proAtS40.4:GUS* seedlings with or without ABA treatment. The 3-day-old seedlings grown on 0.5 × MS medium were treated with 10 μM ABA or mock treatment solution for 6 h before GUS staining. This experiment was repeated three times with similar results. Scale bars, 200 μm. **(C)** Subcellular localization of AtS40.4 in root tip cells of *ats40.4* seedlings expressing *proAtS40.4:AtS40.4-GFP*. Seedling roots were immersed into 100 μM ABA for 0, 2, or 4 h before observation or were transferred onto 24% PEG-6000 (−0.75 Mpa) medium for further growth 1 day as moderate drought stress. Nuclei were labeled with DAPI (shown in a pseudo color representation). Scale bars, 20 μm. **(D)** Numbers of AtS40.4-GFP particles in a root tip cell of seedlings with free or 100 μM ABA, and 24% PEG-6000 applications. Data are means ± SEs based on more than 10 roots each treatment. Double asterisks denote statistical differences with Student’s *t*-test at *P* < 0.01.

Considering that *AtS40.4* functions in ABA signal during early seedling development, we investigated whether the expression of *AtS40.4* was regulated by ABA by using qRT-PCR. Five-day-old wild-type seedlings were placed into 0.5 × MS liquid medium with ABA (10 μM, in ethanol), and the ethanol-treatment as a negative control. As shown in Figure 4A, the expression levels of *AtS40.4* increased to 12- and 36-fold after ABA treatment for 1 and 6 h, respectively. The transgenic lines expressing *proAtS40.4:GUS* were next used to explore the response of *AtS40.4* to exogenous ABA. In the 3-day-old seedlings of the transgenic line, the GUS signal was detected in the cotyledon, hypocotyl, and root. After 10 μM ABA treatment for 6 h, the GUS reaction in the root of the seedling evidently enhanced (Figure 4B). These results indicate that *AtS40.4* is an ABA-responsive gene.

To elucidate how AtS40.4 plays roles at the cellular level, we investigated the subcellular localization of AtS40.4 in the *ats40.4-1* background plants expressing *proAtS40.4*:*AtS40.4-GFP* construct. The green fluorescent signals of GFP were localized in the cytoplasm and the nucleus of the root tip cells (Figure 4C and Supplementary Figure 4A). These results indicate that the AtS40.4 protein is a cytoplasm-nucleus dual-localized protein.

The dual cytosolic/nuclear localization and involvement in ABA signaling of AtS40.4 prompted us to examine whether the distribution of AtS40.4 between the cytoplasm and the nucleus was regulated by ABA as recent reports which the distribution of nucleocytoplasmic trafficking of WD40 protein 1 and C2-domain ABA-related proteins is regulated by ABA (Qin et al., 2019; Xu et al., 2019). Surprisingly, after 100 μM ABA application to the seedling roots, numerous intracellular fluorescent particles appeared in the root tip cells (Figure 4C and Supplementary Figures 4B,C). Further statistical analysis showed that the GFP-particles in the root tip cells under 100 μM ABA treatment for 2 or 4 h were 6- and 20-fold higher than those in the control (Figure 4D), indicating that the formation of these particles containing AtS40.4 was time dependent.

To investigated whether or not the localization changes of AtS40.4 is caused by the stress response from ABA application, we used PEG-6000 to treatment the seedling root as described previously (Chong et al., 2019). The fluorescent of GFP-particles was rarely detected in the root cells after PEG-induced moderate drought severity (-0.75 MPa, 24% PEG-6000) for 1 day (Figures 4C,D and Supplementary Figure 4D). This indicates that the ABA-induced alteration of AtS40.4 localization patterns is different from the stress imposed by drought. Taken together, these results suggest that *AtS40.4* is a novel ABA-responsive member.

### *Ats40.4* Regulates the Expression of *ABIs* and Functions Upstream of *ABI4*

**FIGURE 5:**
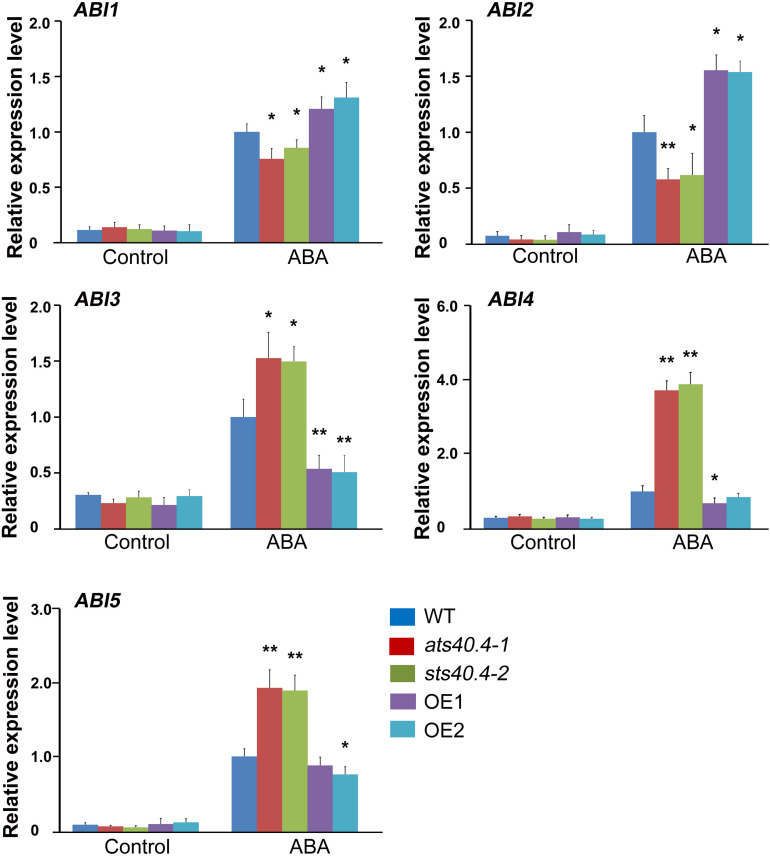
*AtS40.4* affects expression of *ABI1-5.* qR-PCR analysis of the expression levels of *ABI1-5*. The 7-day-old seedlings of WT, *ats40.4* mutants and *AtS40.4*-overexpressing lines (OE-1 and OE-2) were treatment with 0 or 10 μM ABA for 5 h. *GAPDH* was used as the internal control. Each value is the mean ± SE of three biological determinations. Significant differences are denoted with an asterisk (*P* < 0.05) or double asterisks (*P* < 0.01).

Because AtS40.4 was partly localized in the nucleus, we then explored whether AtS40.4 has an impact on gene expression. *ABIs* are core genes in ABA signaling (Leung et al., 1997; Skubacz et al., 2016; Chandrasekaran et al., 2020). Therefore, we tested the transcription levels of these genes in the *ats40.4* mutants and *AtS40.4*-overexpressing lines with and without ABA treatment. The expression levels of *ABI1* and *ABI2* were downregulated in the a*ts40.4* seedlings but upregulated in the *AtS40.4*-overexpressing lines after 10 μM ABA application in roots for 5 h (Figure 5). By contrast, the expression levels of *ABI3*-*5* were enhanced in the a*ts40.4* mutants but decreased in the overexpressing lines. In addition, the expression level of *SnRK2.6*, another core gene in ABA signaling, was increased in a*ts40.4* mutants while decreased in *AtS40.4*-overexpressing lines after ABA treatment (Supplementary Figure 5). We also tested the expression of *RD29A*, an important gene response to ABA and abiotic stress such as drought and salt. The promoter region of *RD29A* contains two cis-acting regulatory elements, an ABRE and two DREs (Narusaka et al., 2003). The transcription level of *RD29A* was promoted by ABA in the *AtS40.4*-overexpressing line, whereas no significant change was detected in the a*ts40.4* mutant (Supplementary Figure 5).

**FIGURE 6:**
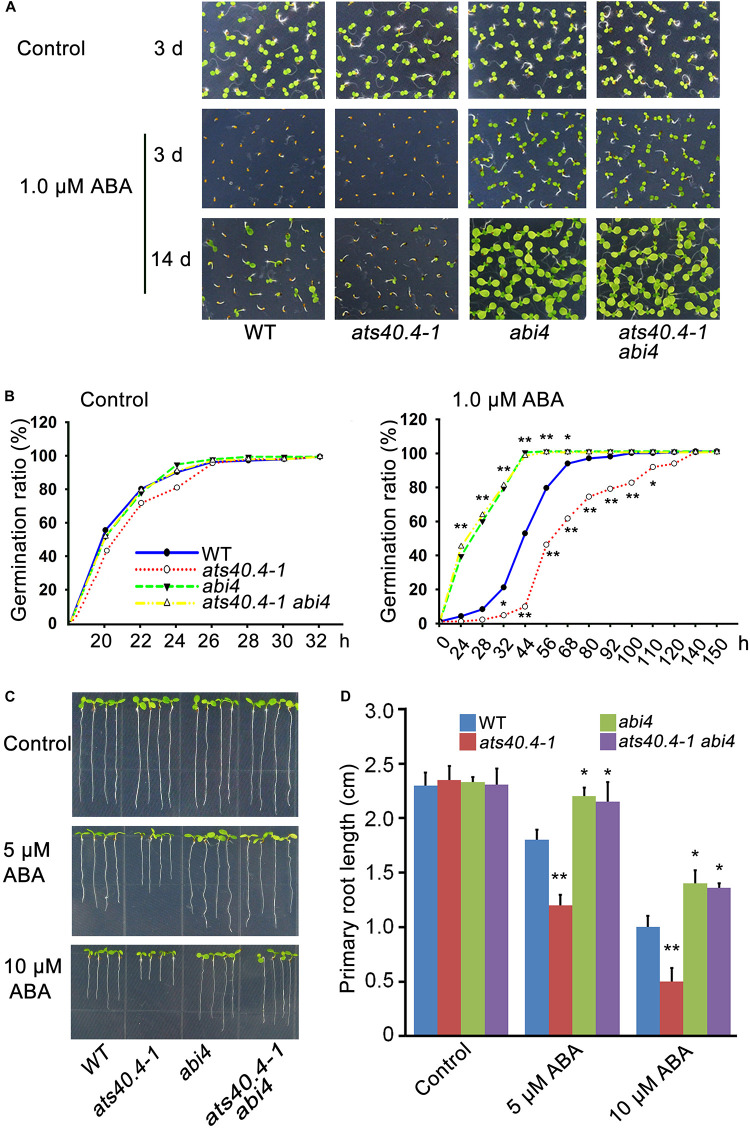
*AtS40.4* functions upstream of *ABI4* in ABA signaling. **(A)** Seed germination and seedling growth of wild type, *ats40.4-1* and *abi4* single mutants, and *ats40.4-1 abi4* double mutant. Seeds were geminated and grown on 0.5 × MS medium containing 0, and 1.0 μM ABA. **(B)** Seed germination ratios of wild type, *ats40.4-1*, *abi4* and *ats40.4-1 abi4* mutants. Seeds were grown on 0.5 × MS medium containing 0 (left) and 1.0 μM ABA (right). The asterisks indicate a significant difference of mutants from the wild-type *Arabidopsis* (Student’s *t*-test; *P < 0.05; **P < 0.01). **(C,D)** Root elongation of seedlings of wild-type, *ats40.4-1, abi4*, and *ats40.4-1 abi4* mutants. The 3-day-old seedlings grown on 0.5 × MS medium were transferred onto the 0, 5, and 10 μM ABA medium for further growth 7 days. **(C)**. Values are means ± SE of three independent biological replicates. Significant differences **(D)** are denoted with an asterisk (*P* < 0.05) or double asterisks (*P* < 0.01).

*AtS40.4* mutations led to a large increment of *ABI4* transcription, indicating that *ABI4* is an important target gene in the *AtS40.4* response to ABA signaling. To further verify the genetic relationship between *AtS40.4* and *ABI4*, we introduced *AtS40.4* mutation into the *abi4* mutant (SALK_080095) and generated the *ats40.4-1 abi4* double mutant. Then, the ABA sensitivity of these different genotypes was investigated. No significant difference was found among the *ats40.4-1 abi4* double mutant, *ats40.4-1* and *abi4* single mutants, and wild type during seed germination and root elongation on 0.5 × MS medium (Figures 6A,B). The *ats40.4-1 abi4* double mutant showed less sensitivity to ABA similar to that of the *abi4* mutant when seeds were germinated on ABA medium (Figures 6A,B). Moreover, the seedlings of the *ats40.4-1 abi4* double mutant displayed an ABA-hyposensitive phenotype as *abi4* seedlings in the presence of ABA (Figures 6C,D). These results indicate that the disruption of *ABI4* abolishes the negative role of *AtS40.4* in ABA signaling, supporting that *ABI4* is genetically epistatic to *AtS40.4* in the ABA-regulated seed germination and early seedling growth.

## Discussion

The role of *AtS40.4* in leaf senescence has been previously characterized in *Arabidopsis* (Fischer-Kilbienski et al., 2010). In this study, we found a novel function of AtS40.4 which acts as a negative regulator in ABA signaling during seed germination and early seedling growth. *AtS40.4* is globally expressed in *Arabidopsis* including the seedlings, both developing and germinated seeds. Moreover, expression of *AtS40.4* is dramatically promoted by ABA in early seedlings, especially in the root cells.

AtS40.4 functions in ABRE-dependent ABA signaling and affects transcription of *ABIs*, *SnRK2.6* and *RD29A*. Interestingly, AtS40.4 enhanced the expression *ABI1* and *ABI2*, two negative regulators of ABA signaling; whereas inhibited the transcription levels of *ABI3-5* and *SnRK2.6*. These results support that AtS40.4 acts as an upstream regulator of ABA signaling and indirectly affects expression of the key genes in ABA signaling. The core negative components ABI1 and ABI2, and positive factors ABI3-5 play diverse roles in the ABA- regulated plant growth and development (Leung et al., 1997; Wind et al., 2013; Cheng et al., 2014; Li P. C. et al., 2016; Gong et al., 2020; Xu et al., 2020). Especially, ABI4, as a transcription factor, either activates or represses multigene expression in ABA signaling (Baek et al., 2020; Chandrasekaran et al., 2020). Moreover, ABI4 acts as a node of integration for different signals, including environmental stimuli, sugar sense, and various phytohormones (Wind et al., 2013; Chandrasekaran et al., 2020). Therefore, the expression regulation of *ABI4* is important for plant growth, development, and response to environmental cues. Several regulators of *ABI4*, such as WRKY and MYB transcription factors, promote or inhibit *ABI4* expression during seed maturation and early seedling development (Cui et al., 2013; Yan et al., 2014; Huang et al., 2016; Phukan et al., 2016; Mu et al., 2017; Ma et al., 2019; Zhao et al., 2019). Our findings reveal that AtS40.4 is a novel upstream regulator of *ABI4* expression.

AtS40.4 is partly localized in the nucleus, consisting with its functions in effecting expression of *ABIs*. AtS40.3 and HvS40 exhibiting nuclear localization have been assigned as DNA binding proteins (Krupinska et al., 2002; Fischer-Kilbienski et al., 2010). Among the nuclear S40 proteins, the probability of AtS40.4 acting as a DNA-binding protein (76%) is lower than that of AtS40.3 (99.5%), HvS40 (95.7%), OsS40-1 (97.4%), and OsS40-14 (88.4%), but higher than OsS40-13 (33.2%) (Supplementary Figures 6, 7) (DNA Binding Protein Prediction)^1^. However, the effect of AtS40.4 on the expression between negative regulators of ABA signaling, *ABI1*-*2*, and positive factors of ABA signaling, *ABI3-5* and *SnRK2.6*, is opposite. This indicates that AtS40.4 does not regulated directly gene transcription although it has a role of binding to DNA. This study, together with the previous findings, is not sufficient to explain functions of S40 proteins in the nucleus. Therefore, our work has just laid the groundwork for further exploration of precise biological roles and mechanism of S40 members in the nucleus.

The localization and partitioning of AtS40.4 are regulated by ABA. Similar result has been previously reported on *Arabidopsis* RopGEF1, a guanine nucleotide exchange factor protein, which plays an important role in the ABA-mediated growth regulation of root. RopGEF1 is rapidly sequestered to intracellular particles after exogenous ABA application, which results in ABA-mediated degradation (Li P. C. et al., 2016). Recent reports have indicated that the nucleocytoplasmic distribution of XPO1-interacting WD40 protein 1 (XIW1) and C2-domain ABA-related (CAR) proteins is also regulated by ABA (Qin et al., 2019; Xu et al., 2019). XIW1 interacts with ABI5 in the nucleus and positively regulates the ABA response in *Arabidopsis.* CAR proteins play roles in ABA response via recruiting ABA receptors to PM to facilitate ABA signaling (Diaz et al., 2016; Qin et al., 2019). The dynamic localization and stability of CAR proteins are regulated by LOWER TEMPERATURE 1 (LOT1) (Qin et al., 2019). ABA promotes XIW1 accumulation in the nucleus, leading to a decreased distribution of XIW1 in the cytoplasm (Xu et al., 2019); whereas ABA reduces the CAR9–LOT1 interaction in the nucleus, resulting in the increased localization of CAR9 to the plasma membrane (Qin et al., 2019). Therefore, the regulatory ways of spatial distribution of the ABA-response proteins are diverse. The small protein AtS40.4 is partitioned into intracellular particles after ABA application, implying that AtS40.4 distribution is rapidly responsive to the ABA. Moreover, the different localization changes of AtS40.4 after ABA and PEG-6000 treatments reveal that AtS40.4 response to ABA is distinct from that of PEG-induced drought stress. Further exploration of the regulation mechanisms of AtS40.4 intro-cellular distribution will shed new light on the molecular characteristics of these DUF584 proteins.

## Data Availability Statement

The original contributions presented in the study are included in the article/Supplementary Material, further inquiries can be directed to the corresponding author/s.

## Author Contributions

X-LW, X-PS, and J-JR conceived and designed the research. X-PS and J-JR performed the experiments with the help from D-FL, H-DQ, YL, and Y-YW. X-LW and X-PS wrote the manuscript with contributions of L-JK. All authors reviewed and approved the final version.

## Conflict of Interest

D-FL was employed by the company Shandong Lufeng Group Co., Ltd. The remaining authors declare that the research was conducted in the absence of any commercial or financial relationships that could be construed as a potential conflict of interest.

## Footnotes

1 https://dnabind.szialab.org/
