# Supplementary material for: Plant-Specific AtS40.4 Acts as a Negative Regulator in Abscisic Acid Signaling During Seed Germination and Seedling Growth in Arabidopsis
Source: Front Plant Sci. 2021 Feb 4;12:622201. doi: 10.3389/fpls.2021.622201 (PMC7889505; doi:10.3389/fpls.2021.622201)
Supplement: Supplementary file 1 [file Data_Sheet_1.PDF]

Supplementary Figures

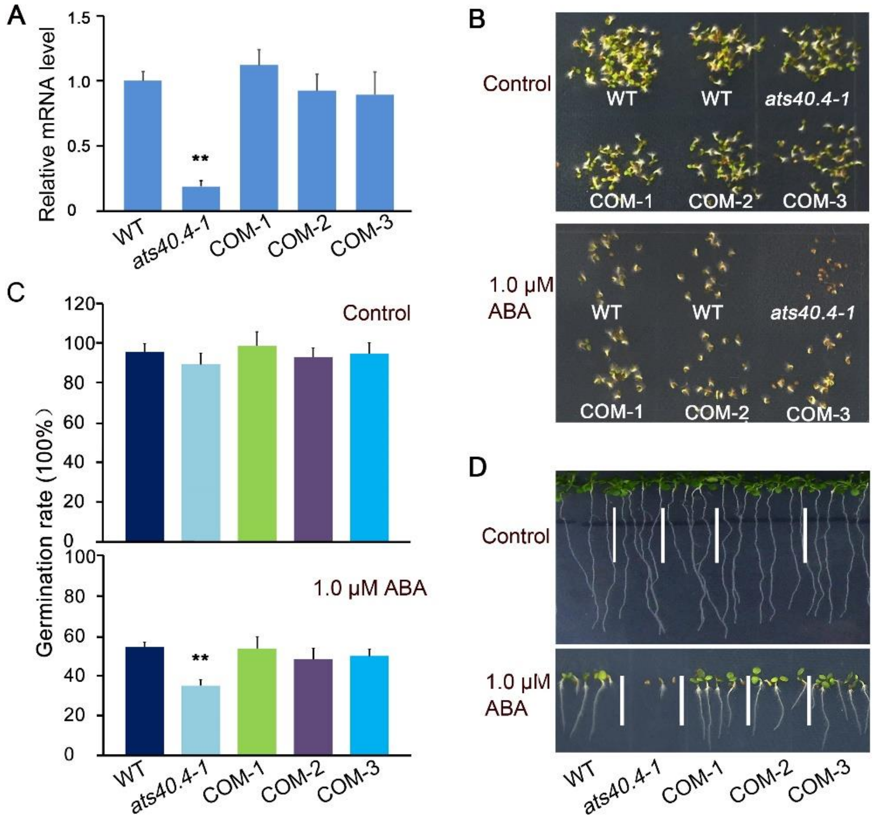

Supplemental Figure 1 *AtS40.4* CDS rescued the increased sensitivity of *ats40.4* mutants to ABA.

(A) The transcription level of *AtS40.4* in wild types Col-0 (WT), *ats40.4-1*, and three rescued lines (COM-1, COM-2 and COM-3). The 10-day-old seedlings were used to detect. *GAPDH* was used as an internal control. Error bars indicate the SEs of three biological replicates. Asterisks denote a significant tatistical difference (Student's *t*-test; **,P < 0.01).

(B) Seed germination of the wild types WT, *ats40.4-1* and three rescued lines grown on 0.5 × MS medium or medium containing 1.0 μM ABA.

(C) Germination rate of seeds grown on 0.5 × MS medium with and without ABA. Double asterisks indicate a significant difference from the control value (Student's *t*-test; **, P < 0.01).

(D) Early seedling growth of WT, *ats40.4* mutants and rescued lines grown on 0.5 × MS medium with 0 and 1.0 μM ABA for germination and growth.

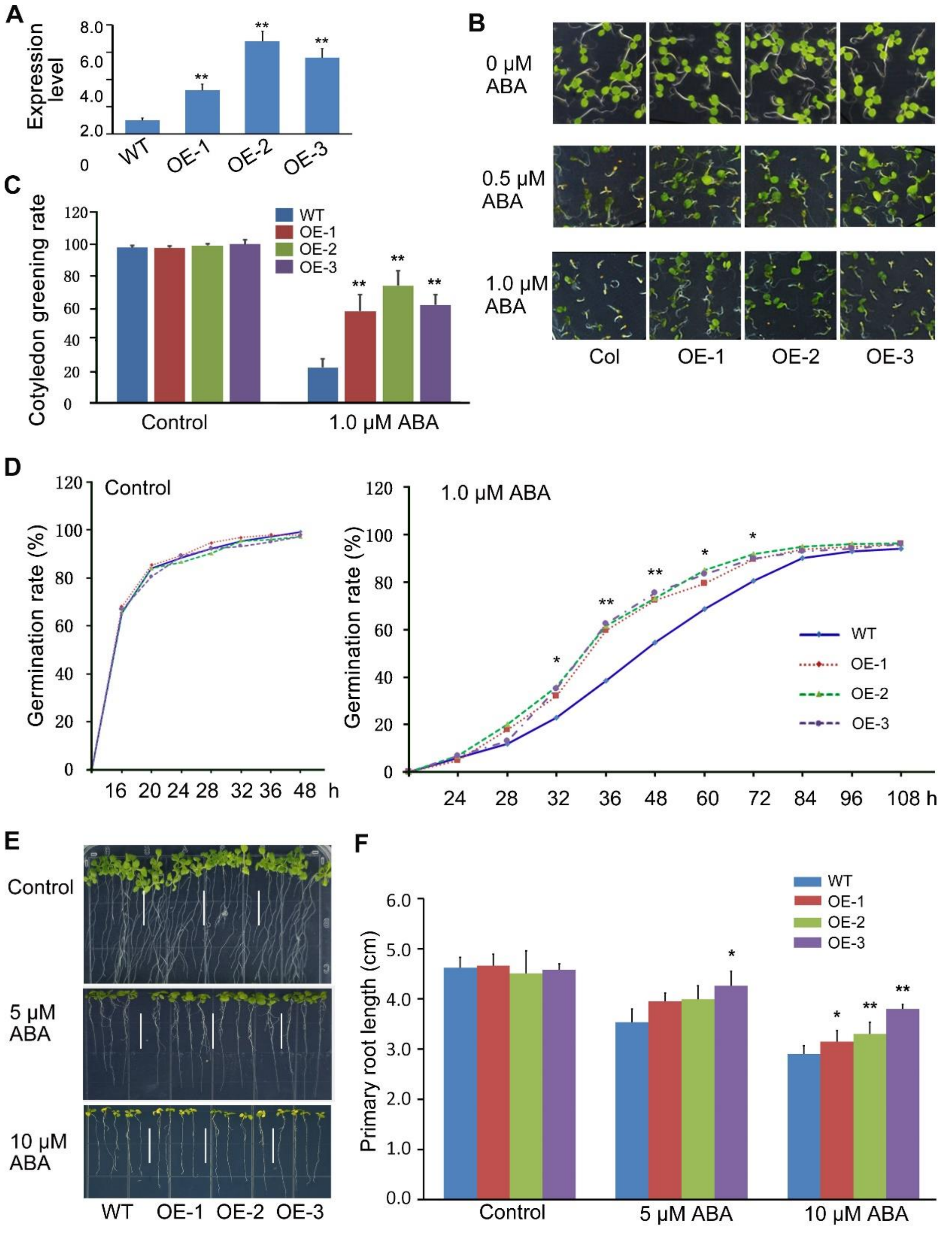

Supplemental Figure 2 Overexpression of *AtS40.4* decreased the ABA sensitivity during seed germination and seedling growth

(A) qRT-PCR analysis of the *AtS40.4* expression level in seedlings of *AtS40.4*-overexpressing lines. *GAPDH* was used as the internal control. Significant differences are denoted with double asterisks (Student's *t*-test; **, $P < 0.01$).

(B) Seedling growth of WT and *AtS40.4* overexpressing lines grown on 0.5 × MS medium containing 0, 0.5 and 1.0 μM ABA.

(C) Cotyledon greening rates of WT and *AtS40.4*-overexpressing seedlings grown on 0.5 × MS medium containing 0 and 1.0 μM ABA. Error bars indicate the SEs of three replicated experiments. Asterisks indicate a significant difference (Student's *t*-test; **, $P < 0.01$).

(D) Seed germination rates of WT and *AtS40.4* overexpressing lines grown on 0.5 × MS medium containing 0 and 1.0 μM ABA. Asterisks indicate a significant difference from the control value (Student's *t*-test; *, $P < 0.05$; **, $P < 0.01$).

(E, F) Root elongation of WT and *AtS40.4*-overexpressing lines. The 3-day-old seedlings grown on 0.5 × MS medium were transferred onto the medium containing 0, 5 and 10 μM ABA for further growth 14 days **(E)**. Data are means ± SEs based on three biological replicates **(F)**. Single and double asterisks indicate statistically significant differences between the overexpressing lines and WT calculated by Student's *t*-test (*, $P < 0.05$ and **, $P < 0.01$).

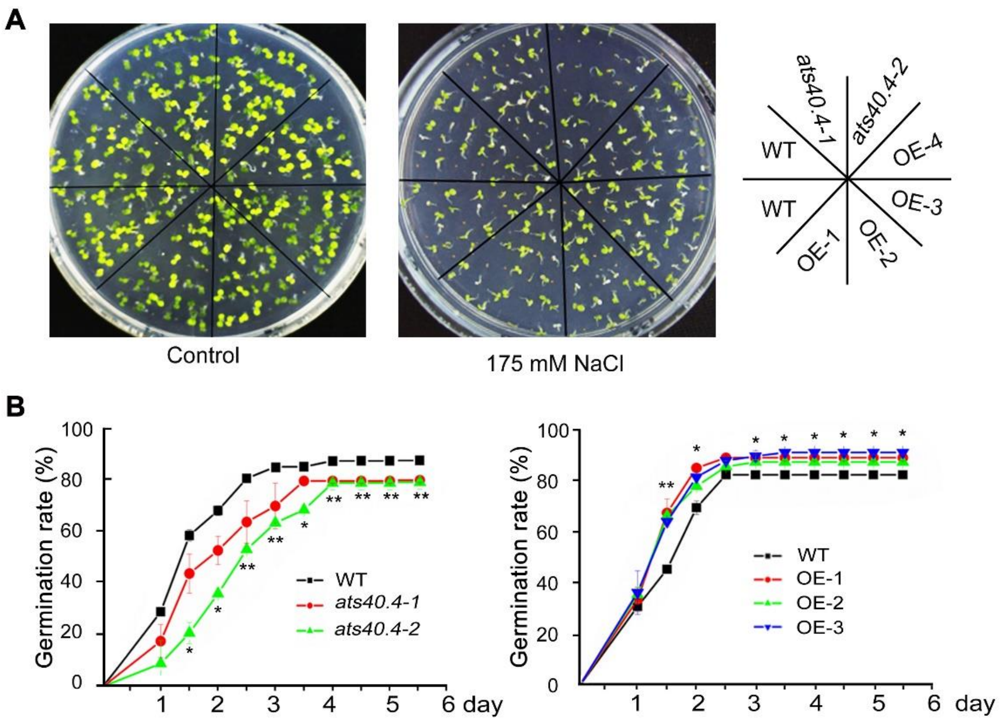

Supplemental Figure 3 *AtS40.4* participates in salt tolerance during seed germination and seedling growth.

(A) Seed germination of the WT, *ats40.4* mutants and *AtS40.4*-overexpressing lines grown on 0.5 × MS medium with or without NaCl.

(B) Seed germination rate of WT, *ats40.4* mutants and *AtS40.4*-overexpressing lines grown on 0.5 × MS medium with 175 mM NaCl. Single and double asterisks indicate significant differences (Student's *t*-test; *, $P < 0.05$ and **, $P < 0.01$).

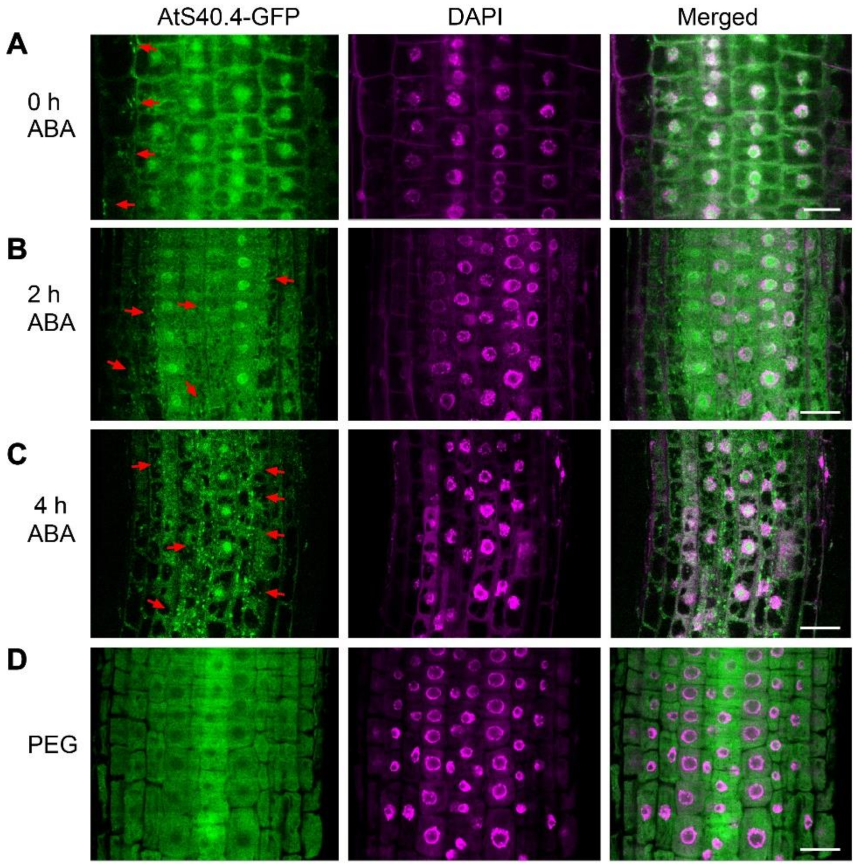

Supplemental Figure 4 Subcellular localization of AtS40.4.

Subcellular localization of AtS40.4 in root tip cells of *ats40.4* background seedlings expressing *proAtS40.4::AtS40.4-GFP* construct. Seedling roots were immersed into 100 μM ABA for 0, 2 or 4 hours or transferred on 0.5 × MS medium containing 24% PEG (-0.75Mpa) for further growth 1 day as moderate drought stress. The nuclei were labeled with DAPI (shown in a pseudo color representation). The arrows indicated GFP-particles in cytoplasm. Scale bars, 20 μm.

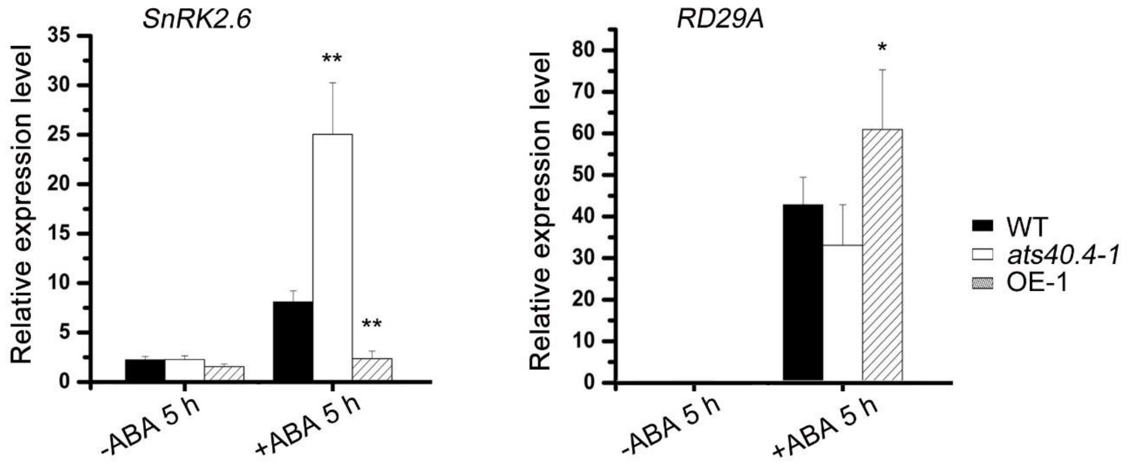

Supplemental Figure 5 *AtS40.4* affects expression of *SnRK2.6* and *RD29A.*

qR-PCR analysis of the expression levels of *SnRK2.6* and *RD29A* in the 7-day-old seedlings of WT, *ats40.4* mutant and *AtS40.4*-overexpressing line. Seedlings were treatment with and without ABA (100 μM) for 5 hours. Each value is the mean ± SE of three independent experiments. Single and double asterisks denote significant differences ($P < 0.05$; $P < 0.01$).

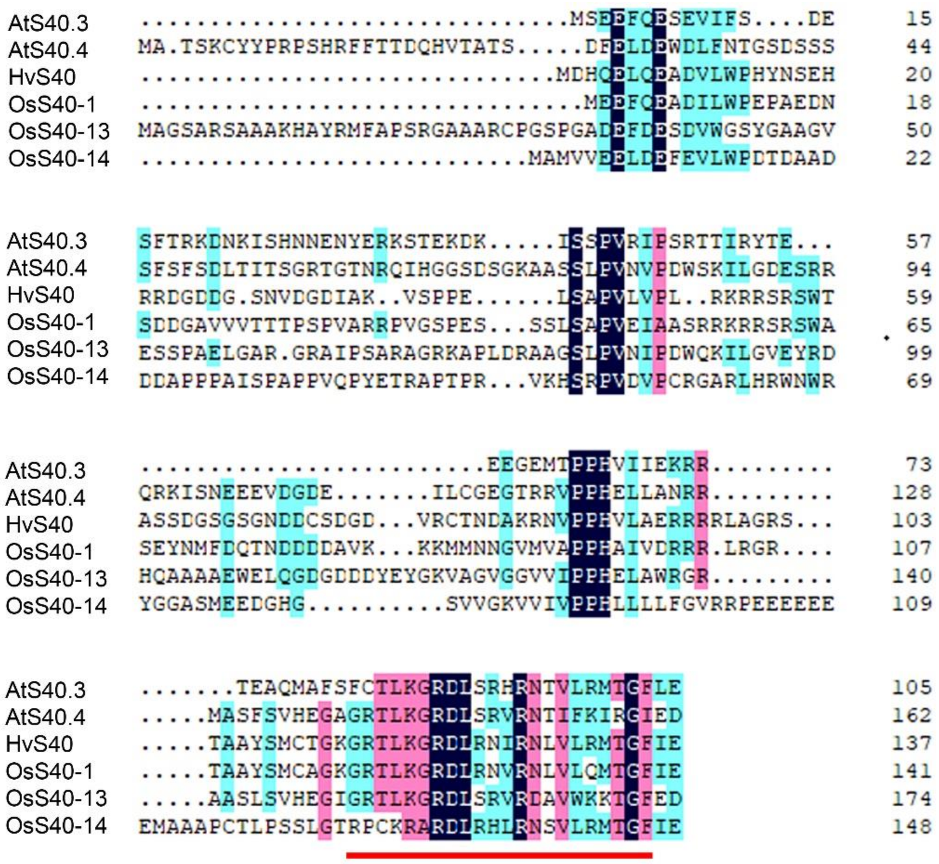

Supplemental Figure 6 Domain assay of nuclear localization S40 proteins. Domain assay of AtS40.3, AtS40.4, HvS40, OsS40-1, OsS40-13 and OsS40-14 proteins with DNAMAN 8, showing the conserved C-terminal DUF584 (red line).

DNABIND prediction

Prediction parameters:

The prediction is done from sequence. The false positive rate is set to 15.0%
Expected sensitivity: 78.3%
Expected Matthews correlation coefficient: 0.63
The score threshold is set to 0.362 (threshold probability: 0.5896)

AtS40.4

Name	Score	Probability of DNA binding	Prediction
Submitted_sequence	1.180	0.7649	DNA-binding

AtS40.3

Name	Score	Probability of DNA binding	Prediction
Submitted_sequence	5.383	0.9954	DNA-binding

HvS40

Name	Score	Probability of DNA binding	Prediction
Submitted_sequence	3.099	0.9569	DNA-binding

OsS40-1

Name	Score	Probability of DNA binding	Prediction
Submitted_sequence	3.638	0.9744	DNA-binding

OsS40-13

Name	Score	Probability of DNA binding	Prediction
Submitted_sequence	-0.700	0.3319	non-DNA-binding

OsS40-14

Name	Score	Probability of DNA binding	Prediction
Submitted_sequence	2.034	0.8844	DNA-binding

Supplemental Figure 7 DNA-binding prediction of S40 family members localized in the nucleus.

This analysis was performed of AtS40.3, AtS40.3, HvS40, OsS40-1, OsS40-13 and OsS40-14 proteins by utilizing bioinformatics web DNABIND prediction (https://dnabind.sziala b.org/).
