# Supplementary material for: Plant-Specific AtS40.4 Acts as a Negative Regulator in Abscisic Acid Signaling During Seed Germination and Seedling Growth in Arabidopsis
Source: Front Plant Sci. 2021 Feb 4;12:622201. doi: 10.3389/fpls.2021.622201 (PMC7889505; doi:10.3389/fpls.2021.622201)
Supplement: Supplementary file 2 [file Data_Sheet_2.PDF]

Supplementary Table S1-S4 Primers used in this study.

Table S1 Primers used for identification of *ats40.4* mutants. LP, forward primer; RP, reverse primer.

**Primer**	**Sequence**	**Mutant**
ats40.4-1LP	5'-TAAAACTCGGAAAGGATGCG C-3'	GABI_561G08
ats40.4-1RP	5'-AAACGAAATTCAAGTTTTGAGC-3'	
ats40.4-2LP	5'-CGTCAACTTCTTCCTCATTCG-3'	GABI_863C05
ats40.4-2RP	5'-CATTCGTCTAGCTCGAAATCG -3'	
LBGABI	5'- ATATTGACCATCATACTCATTGC-3'	For GABI mutants

Table S2 Lists of primers used for vector construction and identification of transgenic lines. F, forward primer; R, reverse primer.

**Primer**	**Sequence**	**Amplicon Size and Construct**
AtS40.4F_ Xba I	5'-TCTAGATTCTTCTTCTTGAAATGGC-3'	506 bp including the *AtS40.4* CDS (489 bp) and 17 bp UTR;
AtS40.4R_ Kpn I	5'-GGTACCTAATTAATCTTCGATCCCTC-3'	*35S::AtS40.4*
AtS40.4PF	5'-GTTCAACCAAAACTCTTACCTTCA-3'	1403 bp promoter, 489 bp CDS, and 3 bp 3′ UTR of *AtS40.4*;
AtS40.4R	5'-TAAATAATCTTCGATCCCTC-3'	*proAtS40.4::S40.4-GFP*
AtS40.4PF	5'-GTTCAACCAAAACTCTTACCTTCA-3'	1403 bp promoter of *AtS40.4*;
AtS40.4PR	5'-GGCGGTGACGTGTTGGT-3'	*proS40.4::GUS*
35SF	5'-GAAGGTGGCTCCTACAAATGCCA -3'	Together with *AtS40.4R_KpnI* for identification of *35S::AtS40.4* transgenic lines
GUSR	5'-CACAGTTCATAGAGATAACCTTCACCCG - 3'	Together with *AtS40.4PF* for identification of *proS40.4::GUS* transgenic plants
GFPR	5'-CACCTTCACCCTCTCCACTG -3'	Together with *AtS40.4PF* for identification of *proAtS40.4::S40.4-GFP* transgenic lines

The boxes in the primer sequences indicate the star and terminal codons of *AtS40.4* CDS. The underlines denote the nucleotide sequence for exonucleases *Xba I* and *Kpn I*. The underline and box indicate the mutated terminal codons of *AtS40.4* CDS.

Table S3 Primers used for RT-PCR analysis for *AtS40.4* expression level. F, forward primer; R, reverse primer.

Primer	Sequence	Gene
AtS40.4RTF	5'-ATGGCGACGAGCAAGTGCTA-3'	*AtS40.4*
AtS40.4RTR	5'- ATCTTCGATCCCTCTAATTTTAAAA -3'	(486bp)
TUBULIN2F	5'-GGTATCCAGGTCGGAAATGC-3'	Internal control gene
TUBULIN2R	5'-TCCCGTAGTCAACAGAAAGT-3'	*TUBULIN2*

The boxes in the primer sequences indicate the star codons of *AtS40.4* CDS. The PT-PCR product includes the sequences of *AtS40.4* CDS from star codons to the nucleotide sequence before terminal codons TAA of AtS40.4 CDS.

Table S4 Primers used for qRT-PCR analysis. F, forward primer; R, reverse primer.

Primer	Sequence	Gene
AtS40.4qRTF	5'-GCCACCGTTTCTTCACCAC-3'	*AtS40.4*
AtS40.4qRTR	5'-CGGATGTGATTGTAAGGTCACTAAAG-3'	(132 bp)
ABI1F	5'-CCATGTCGAGATCCATTGGC-3'	*ABI1*
ABI1R	5'-CAGTTCAAGGGTTTGCTCTTGAG-3'	
ABI2F	5'-ATGGCGGTTCTCAGGTAGCGA-3'	*ABI2*
ABI2R	5'-AACCACCGAGGTAGACCCAACAG-3'	
ABI3F	5'-ACGTCAGCAGGTGGTACCAG-3'	*ABI3*
ABI3R	5'-GGCAAGTGTGTCTCAGCTTC-3'	
ABI4F	5'-ATGGACCCTTTAGCTTCCCAAC-3'	*ABI4*
ABI4R	5'-CTTTGCGTTTGCGTTGAGCG-3'	
ABI5F	5'-TCGACAAGGCTCTTTGACAC-3'	*ABI5*
ABI5R	5'-ATTACCGCTACCACCACCTC-3'	
RD29AF	5'-GCCGACGGGATTTGACG-3'	*RD29A*
RD29AR	5'-GCCGGAAATTTATCCTCTTCTGA-3'	
SnRK2.6F	5'-TCCGGTAATTTTGGAGTTGC-3'	

SnRK2.6R	5'-CCACCATTAATACCACGTTCC-3'	*SnRK2.6R*
GAPDHF	5'-TGGTTGATCTCGTTGTGCAGGTCTC-3'	*GAPDHR*
GAPDHR	5'-GTCAGCCAAGTCAACAACTCTCTG-3'	
